# First Report of *Myocastor coypus* Infected with *Staphylococcus cohnii*

**DOI:** 10.1155/2024/3710299

**Published:** 2024-03-27

**Authors:** Wei Dong, Nana Peng, Lei Yang, Huimin Ning, Jie Fan, Xinying Li, Yuhao Chen, Xu Han, Meng Ge

**Affiliations:** ^1^College of Veterinary Medicine, Hunan Agricultural University, Changsha, China; ^2^Hunan Engineering Technology Research Center of Veterinary Drugs, College of Veterinary Medicine, Hunan Agricultural University, Changsha, China

## Abstract

In December 2021, a wildlife conservation base in Hunan, China, reported illness and death among its captive *Myocastor coypus* population. A gram-positive pathogen was isolated from the affected animals. The 16S rDNA sequence of the isolated strain was approximately 1,500 bp long and exhibited 98.4% homogeneity with *Staphylococcus cohnii* found in sea cucumbers. Interestingly, the biochemical reactions of the isolated strain were consistent with the characteristics of *S. cohnii*. The LD100 for BALB/c mice was 1.2 × 10^7^ CFU/g, while the MLD was 0.6 × 10^7^ CFU/g. Inflammatory cell infiltration was observed in the liver, spleen, and kidneys of infected mice, accompanied by widespread systemic bacteremia and focal hepatic and splenic necrosis. Moreover, mycelium was detected in the liver and kidney. The isolated strain possessed both HLB and PVL virulence genes. To the best of our knowledge, this is the first study to report a highly pathogenic strain of *S. cohnii* isolated from *M. coypus*, possessing both HLB and PVL virulence genes. The findings contribute to yielding a better understanding of the pathogenic mechanisms of *S. cohnii* and have significant implications for disease control in *M. coypus*, as well as for public health safety and the prevention of zoonotic diseases.

## 1. Introduction

*Myocastor coypus*, also referred to as nutria, suricate, or coypu, belongs to the Rodentia order and the Myocastoridae family. It is a large, semiaquatic rodent indigenous that is currently classified as an endangered species for wildlife conservation [1]. Originally native to South America, *M. coypus* exhibits characteristics such as early sexual maturity, strong reproductive capabilities, tolerance to coarse feed, and adaptability to both aquatic and terrestrial environments [2]. In recent years, artificial breeding has been initiated to fulfill nonedible demands for conservation, medicinal use, exhibition, and scientific research. With the expansion of the breeding scale and increased stocking density, diseases among captive *M. coypus* are also on the rise.

*Staphylococcus cohnii* was discovered in 1957 by Schleifer and Kloos and subsequently named by German bacteriologist Ferdinand Cohn. It is a conditional pathogen, and the majority of isolated strains have been identified in humans and other primates [3, 4]. Notably, the bacterium has also been isolated from goats, dogs, cows, ducks, pigs, bees, *Culex quinquefasciatus* mosquitoes, and brown rats [5–12]. In recent years, there has been an increasing number of reports on human infections caused by *S. cohnii* [3], primarily leading to conditions such as bacteremia, acute cholecystitis, brain abscess, infective endocarditis, and pneumonia [13–15]. To date, there have been no reports of *M. coypus* infected with *S. cohnii*.

In December 2021, a wildlife conservation base in Hunan province, China, experienced a sudden outbreak of illness among its *M. coypus* population. The symptoms included elevated body temperature, lethargy, and decreased food intake, leading to several fatalities. Thereafter, the conservation base dispatched the deceased *M. coypus* to a laboratory for autopsy, pathogen isolation and identification, biochemical testing, 16S rDNA sequencing analysis, virulence gene analysis, and histopathological examinations. The diagnosis confirmed that the animal was infected by *S. cohnii*.

## 2. Materials and Methods

### 2.1. Sample

A wildlife conservation base in Hunan, China, dispatched the deceased *M. coypus* preserved at low temperatures to the laboratory for autopsy and aseptic collection of liver and spleen samples.

### 2.2. Experimental Animals

Male BALB/c mice, procured from Hunan Silaikejingda Experimental Animal Co., Ltd. and weighing 20 ± 2 g, were used in this study.

### 2.3. Isolation, Purification, and Gram Staining of Bacteria

Liver and spleen tissues were aseptically collected and inoculated onto a standard broth medium (5% FCS) at 37°C for 12 hr. Then, the cultured bacterial fluid was then streaked onto blood agar plates and incubated at 37°C for another 12 hr. Single colonies were isolated, gram-stained, and further purified.

### 2.4. 16S rDNA Sequencing and Homology Analysis of Isolated Strains

Universal primers for amplifying bacterial 16S rDNA gene sequences were synthesized according to the reference literature [16]. The primers, namely 16S rDNA-27F: 5-AGAGTTTGATCCTGGCTCAG-3 and 16S rDNA-1492R: 5-GGCTACCTTGTTACGACTT-3, were synthesized by Beijing Qingke Biotechnology Co., Ltd.

The isolated bacterial strain was inoculated onto a standard broth medium and incubated at 37°C for 12 hr. An appropriate amount of bacterial fluid was collected for genomic DNA extraction, following the specific steps outlined in the instruction manual of the relevant reagent kit (from Takara Company). Using the extracted genomic DNA of the bacterial strain as a template, the 16S rDNA gene was amplified. The PCR system consisted of a 2 × PCR master mix (25 *μ*L), 1 *μ*L of each upstream and downstream primer, 3 *μ*L of DNA template, and 20 *μ*L of double-distilled water. The amplification conditions were as follows: initial denaturation at 94°C for 5 min, followed by 35 cycles of 95°C for 30 s, 56°C for 30 s, and 72°C for 90 s; and a final extension at 72°C for 10 min. 8 *μ*L of the PCR product was subjected to 1% agarose gel electrophoresis. The PCR-positive products were sent to Beijing Qingke Biotechnology Co., Ltd. for sequencing.

Next, the sequencing results were compared with reference strains in the NCBI database using Blast homology analysis. Additionally, a phylogenetic tree was constructed using the Neighbor Joining method in MEGA 6.0 software.

### 2.5. Biochemical Tests

Attributed to the high 16S rDNA gene homology between the isolated strain and *S. cohnii*, biochemical reactions were selected based on the “Bergey's Manual of Systematic Bacteriology.” Microbiochemical reaction tubes were sourced from Hangzhou Microbiology Co., Ltd. The isolated strain was inoculated onto a standard broth medium and incubated at 37°C for 12 hr. The cultured bacterial fluid was subsequently inoculated into selected microbiochemical identification tubes and incubated at 37°C for 48 hr.

### 2.6. Mouse Pathogenicity Test

The isolated bacteria were inoculated into a nutrient broth medium and incubated at 37°C for 12 hr. The concentration of the bacterial fluid was determined using a live bacterial count method. The bacterial fluid was then diluted to various concentrations using sterilized physiological saline. BALB/C mice were divided into six groups, with 10 mice in each group for the *S. cohnii* test, and one control group of 10 mice was injected with sterilized physiological saline. Intraperitoneal injections were administered, and mortality was observed and recorded within 48 hr. The lowest bacterial count that resulted in 100% mortality in mice was considered the absolute lethal dose (LD100) for intraperitoneal infection of the strain. The dose that caused individual mouse deaths and its lower-tier dose that did not result in death was considered the minimum lethal dose (MLD) for intraperitoneal infection of the strain. Postmortem examinations were conducted on the deceased mice in a sterile environment. Pathological tissues were collected, and the pathogenic bacteria were isolated, purified, and cultured using the method described in Section 2.3. Hematoxylin and Eosin (HE) staining was performed to analyze pathological changes. The pathological tissues were soaked in a 10% polyformaldehyde solution, embedded in paraffin, sectioned, stained, and observed under a microscope.

### 2.7. Detection of Virulence Genes

Primer sequences and annealing temperatures are presented in Table 1. Template DNA was extracted using the boiling method. PCR was used to amplify virulence genes of Staphylococcus, including *β*-hemolysin (HLB), leukocidin (PVL), *α*-hemolysin (HLA), *δ*-hemolysin (HLD), coagulase (coa), clumping factor (clfA), and *γ*-hemolysin (hlg) [17, 18]. The relevant primers were synthesized by Beijing Qingke Biotechnology Co. Ltd.

### 2.8. Antimicrobial Susceptibility Testing

According to the Clinical and Laboratory Standards Institute (CLSI), the drug-sensitive paper diffusion method was adopted to determine the susceptibility of the isolated pathogenic bacteria. The antimicrobials subjected to testing were as follows: *β*-lactam antibiotics (amoxicillin, penicillin G, ceftriaxone, cefradine, cephalexin, and ampicillin), quinolones (enrofloxacin and levofloxacin), lincosamides (clindamycin), aminoglycosides (gentamycin, kanamycin, and streptomycin), polypeptide (polymyxin B), nitroimidazoles (metronidazole), sulfonamides (sulfamethoxazole and sulfafurazole), macrolides (erythromycin, roxithromycin, and clarithromycin), and tetracyclines (doxycycline). The results were analyzed in accordance with CLSI standards.

## 3. Results

### 3.1. Bacterial Isolation and Identification Experiment Results

**Figure 1:**
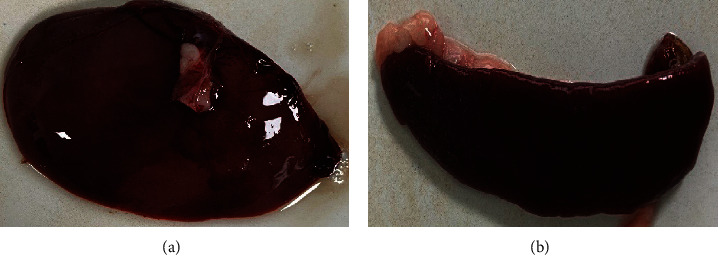
Pathological dissection diagram: (a) liver and (b) spleen.

**Figure 2:**
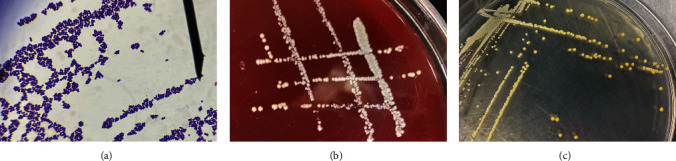
Morphology and staining characteristics of isolated bacteria and colony morphology: (a) gram stain morphological characteristics; (b) blood agar plate colony morphology; and (c) regular culture medium plate colony morphology.

Postmortem examinations of the deceased *M. coypus* revealed mild hepatomegaly (Figure 1(a)) and splenomegaly with blunt edges (Figure 1(b)). Afterward, liver and spleen tissues were inoculated onto blood agar plates, and regular culture medium plates were incubated at 37°C for 12 hr. Smooth, round, white colonies proliferated on the blood agar plates from both liver and spleen tissues without evidence of hemolysis (Figure 2(b)). Yellow colonies with intact edges grew on regular culture medium plates (Figure 2(c)). Single colonies were randomly selected and gram-stained, revealing grape-like clusters of purple cocci (Figure 2(a)).

### 3.2. PCR Amplification Results and Sequence Analysis

**Figure 3:**
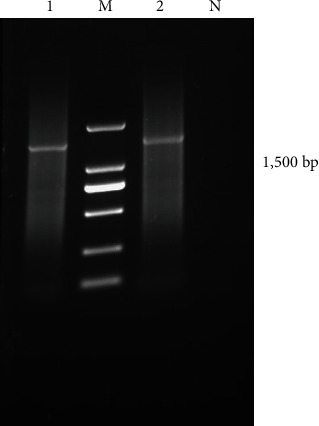
Electrophoresis of 16S rDNA amplification products. 1: 16S rDNA amplification product of spleen-isolated bacteria; 2: 16S rDNA amplification product of liver-isolated bacteria; M: Marker DL2000; N: Negative control.

**Figure 4:**
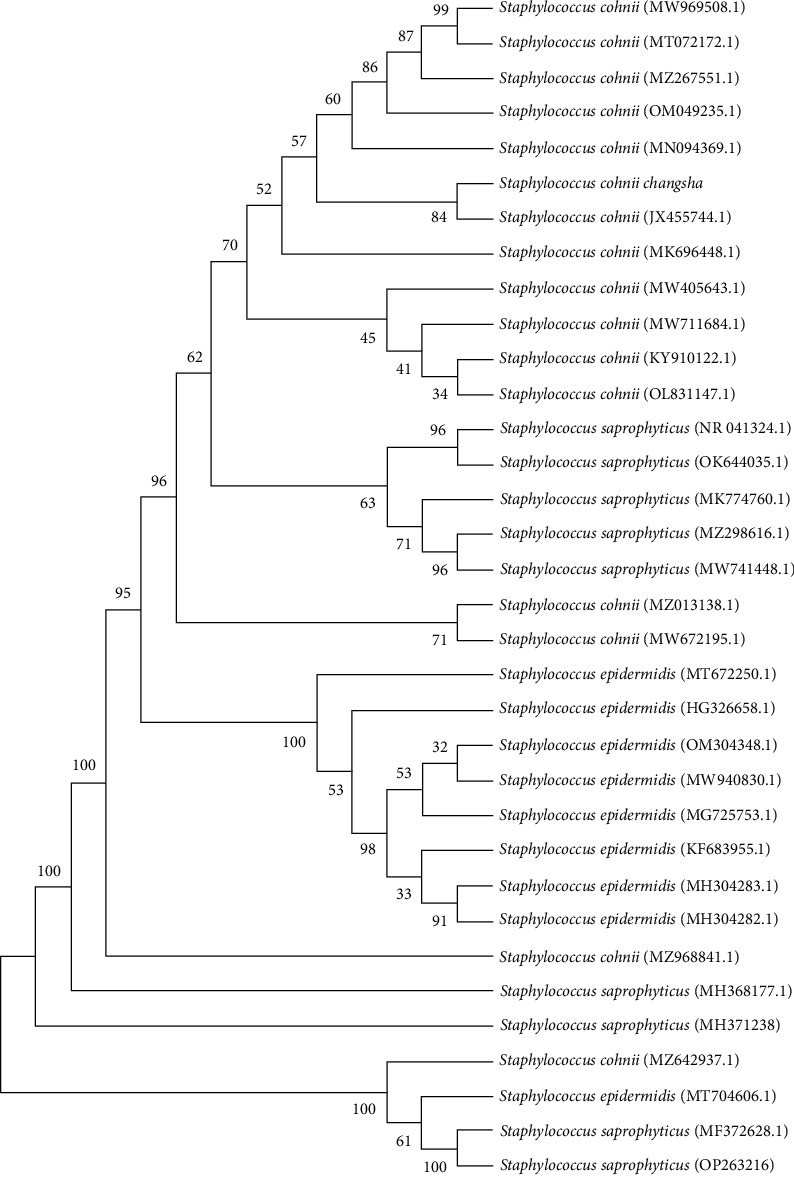
Evolutionary tree of Staphylococcus 16S rDNA gene sequence and genetic evolutionary analysis with related pathogens.

Using the genomic DNA of bacteria isolated from the liver and spleen as templates, the size of the PCR amplification products for 16S rDNA universal primers was determined to be approximately 1,500 bp (Figure 3). As anticipated, the sequencing results of the 16S rDNA gene sequences from liver and spleen isolated bacteria were completely consistent. The homology of the isolated bacterial strain's 16S rDNA gene sequence with that of sea cucumber-derived Staphylococcus reached 98.4% (GeneBank: MK696448.1). Importantly, the isolated strain belongs to the same branch as a known Staphylococcus (JX455744.1; Figure 4). Therefore, it can be inferred that the isolated strain belonged to Staphylococcus originating from *M. coypus*.

### 3.3. Biochemical Test Results

The results of the sugar fermentation tests conducted on the isolated bacteria exposed that glucose, maltose, and mannitol generated acid and gas, whereas lactose and sucrose did not. At the same time, the citrate test results for the isolated bacteria were positive. On the other hand, the results of the indole test, MR test, hydrogen sulfide, and V–P test were negative. Taken together, the biochemical test results of the isolated bacteria were in agreement with the biochemical characteristics of Staphylococcus (Table 2).

### 3.4. Mouse Pathogenicity Test Results

**Figure 5:**
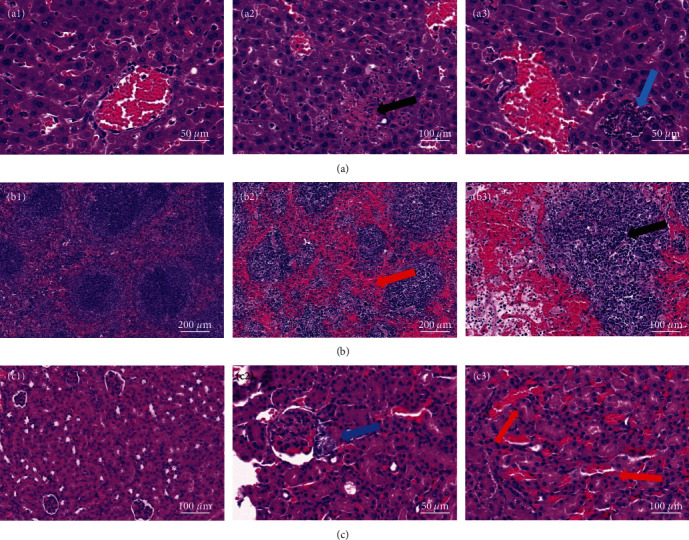
Histological observations of liver, spleen, and kidney tissues in BALB/c mice infected with the isolated bacteria: (a)-a1: normal group liver tissue, a2: in liver tissue, focal necrosis of hepatocytes is visible, with fragmented and dissolved cell nuclei and increased eosinophilic cytoplasm (indicated by black arrows), and a3: hyphae are visible in hepatocytes (indicated by blue arrows); (b)-b1: normal group spleen tissue, b2: in spleen tissue, the white pulp varies in size and shape, and extensive hemorrhages are visible in the red pulp (indicated by red arrows), and b3: extensive focal necrosis is visible within the white pulp (indicated by black arrows); and (c)-c1: normal group kidney tissue, c2: bacterial clumps are noted around renal glomeruli (indicated by blue arrows), and c3: extensive hemorrhagic infiltration is visible in the renal tubular interstitium (indicated by red arrows).

The LD100 (lethal dose causing 100% mortality) for BALB/C mice intraperitoneally infected with the isolated bacteria was 1.2 × 10^7^ CFU/g, whilst the MLD was determined to be 0.6 × 10^7^ CFU/g (Table 3). No abnormalities were observed in the control group of mice. Dead mice presented with hemorrhagic spots and swelling in the liver, spleen, and kidneys. Thus, tissue samples from these organs were streaked onto blood agar plates, and the bacterial culture and morphological staining characteristics were determined to be consistent with those of the isolated bacteria. Likewise, the 16S rDNA gene sequencing results were identical to those of the isolated bacteria. HE staining results displayed significant pathological changes in the liver, spleen, and kidneys of mice infected with the isolated bacteria compared to those in the control group (Figure 5).

### 3.5. Results of Virulence Gene Detection

**Figure 6:**
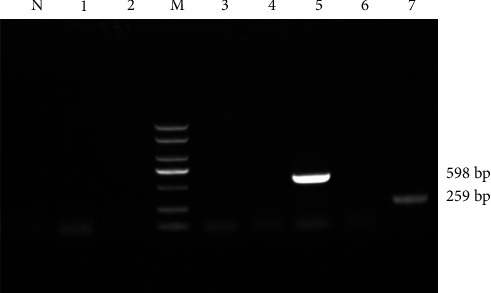
Electrophoresis of amplified virulence genes. N: Negative control; 1: HLA; 2: Hld; 3: coa; 4: clfA; 5: PVL; 6: hlg; 7: HLB; M: Marker DL2000.

The isolated bacterial strain contained staphylococcal virulence genes: Amplification from the genomic DNA of the isolated strain yielded a 259 bp HLB and a 598 bp PVL virulence gene (Figure 6). Contrastingly, no amplification was obtained for HLA, Hld, coa, clfA, and hlg virulence genes.

### 3.6. Antimicrobial Susceptibility Results

The results of the drug resistance test indicated that the strain was sensitive to *β*-lactam, moderately sensitive to polypeptides, tetracyclines, quinolones, and aminoglycosides, and resistant to macrolides, nitroimidazoles, and sulfonamides (Table 4).

## 4. Discussion

In 2016, *M. coypus* was introduced into the IUCN Red List of Threatened Species, prompting wildlife conservation centers both domestically and internationally to focus on its artificial breeding. During this breeding process, cases of infections by pathogens such as *Escherichia coli*, Salmonella, Cryptosporidium, and liver flukes have been reported [19–23]. In December 2021, we isolated a strain of *S. cohnii* from *M. coypus* for the first time, which contained both HLB and PVL virulence genes and had strong pathogenicity. To date, such a highly pathogenic strain of *S. cohnii* affecting *M. coypus* has not been reported in previous research. This study may provide a more comprehensive understanding of *S. cohnii* and its pathogenicity and holds significant reference value for future investigations into its pathogenicity and mechanisms of disease.

*S. cohnii* has long been considered a commensal organism of the skin, capable of effectively suppressing skin inflammation [24]. However, recent years have seen increasing reports of *S. cohnii*-induced infections, leading to the occurrence of various diseases in animals and humans, encompassing sepsis, brain abscess, and pneumonia [25–28]. Nonetheless, animal experimental studies remain scarce. To determine the pathogenicity of the *S. cohnii* strain, it was isolated and then inoculated into mice. The results demonstrated that the LD_100_ of the isolated *S. cohnii* for mice was 1.2 × 10^7^ CFU/g, while the MLD was 0.6 × 10^7^ CFU/g, implying an LD_50_ between 0.6 and 1.2 × 10^7^ CFU/g. This is significantly lower than the lethal doses reported for pathogenic strains such as *Staphylococcus epidermidis* and *Staphylococcus saprophyticus* in mice, whose LD_50_ are 2.7–2.9 × 10^7^ CFU/g and 6–8 × 10^7^ CFU/g, respectively [29], as well as the 11 strains of *S. epidermidis* isolated from humans by Herndon, with LD_50_ ranging from 1.175 × 10^8^ CFU/g to 1.27 × 10^8^ CFU/g for clone groups A and B, and an average of 2.08 × 10^8^ CFU/g for the remaining six strains [30]. Moreover, in terms of pathological features, mice infected with the isolated *S. cohnii* strain manifested pathological changes such as hemorrhagic spots on the surface of the liver, spleen, and kidneys and mild hepatomegaly. These features share similarities with the pathological alterations observed in the infected *M. coypus*. Previous reports on *S. saprophyticus* and *S. epidermidis* infections in mice are typically associated with pathological changes such as splenic, hepatic, and renal abscesses, along with infiltration by neutrophils, eosinophils, and monocytes [29, 31]. However, histopathological examination of mouse tissues infected with the *S. cohnii* strain isolated in this study exposed infiltration by inflammatory cells and widespread systemic sepsis. Additionally, focal necrosis, visible hyphae, nuclear fragmentation, and dissolution, and increased cytoplasmic acidophilia were observed in hepatocytes, whilst irregularly sized and shaped white pulps, focal necrosis, and extensive hemorrhage in the red pulp were detected in splenic tissues, and bacterial clusters appeared around renal glomeruli, with extensive hemorrhagic infiltration in the renal interstitium. The pathogenicity of the *S. cohnii* strain isolated from infected cases of *M. coypus* in our lab differed from that reported for *S. epidermidis* and *S. saprophyticus*. *S. epidermidis* are generally nonpathogenic conditional bacteria that do not synthesize toxins and have weak pathogenicity, while *S. saprophyticus* are conditional pathogens. Therefore, we speculate that the *S. cohnii* strain isolated in this study has stronger pathogenicity in infected mice.

The pathogenicity of staphylococci can be attributed to their ability to express various virulence genes, which are closely related to evasion, invasion, and destruction of host defenses, as well as toxin mediation [32, 33]. Common virulence genes include HLB, PVL, HLA, HLD, coa, clfA, and hlg. HLD was once considered a virulence gene in human pathogenic *S. cohnii* [34]. Nevertheless, recent studies have documented that *Staphylococcus aureus* carrying the PVL virulence gene is more virulent than those lacking it [35]. According to earlier studies, *S. cohnii* has been isolated from ducks, and the presence of three synergistic peptide hemolysins was manually calculated through whole-genome sequencing [6]. The *S. cohnii* strain isolated from *M. coypus* contained both HLB and PVL virulence genes, which is a novel discovery. This finding is hypothesized to be related to the strong pathogenicity of the bacterium. Given the presence of these virulence genes, it is reasonable to speculate that this particular strain has enhanced pathogenic capabilities, potentially posing a greater risk to *M. coypus* and possibly other hosts.

Given the potential risks of *S. cohnii* to humans and animals, it is critical to determine the sensitivity of the strain to drugs. Herein, the drug resistance test determined that the strain was sensitive to *β*-lactams, moderately sensitive to tetracyclines, polypeptides, quinolones, and aminoglycosides, and resistant to macrolides, nitroimidazoles, and sulfonamides, which may serve as a valuable reference for the treatment of *S. cohnii*-induced infections.

Although the case was initially discovered in *M. coypus*, our research signaled that the isolated strain also has high pathogenicity in mice. Considering that *S. cohnii* is a zoonotic disease, the possibility of cross-host infections occurring in mice as well as humans cannot be excluded. The potential infection and pathogenic risks to humans and other animals are indeed a matter of high concern. To prevent *S. cohnii* from posing a threat to public health, it is essential to consider preventive measures in the artificial breeding of *M. coypus*. Given its zoonotic nature, it is vital to implement biosecurity measures to not only protect *M. coypus* but also mitigate the risk of cross-species transmission, thereby safeguarding public health.

## Figures and Tables

**Table 1:** Information on primers for amplifying virulence genes.

Primer name	Primer sequence	Annealing temperature (°C)	PCR product (bp)
HLB-F	5′-GGTACTTGGCCGTTCCACTT-3′	52	259
HLB-R	5′-TTGACCTACTGAGCAGCGTG-3′		
PVL-5	5′-TATTGGTGATGGCGCTGAGG-3′	55	598
PVL-6	5′-ACCACTGTGTACTAATGGGGG-3′		
HLA-F	5′-GCCAAAGCCGAATCTAAG-3′	57	608
HLA-R	5′-GCGATATACATCCCATGGC-3′		
hlg-1	5′- GTCAYAGAGTCCATAATGCATTTAA-3′	53	535
hlg-2	5′-CACCAAATGTATAGCCTAAAGTG-3′		
hld-1	5′-AAGAATTTTTATCTTAATTAAGGAAGGAGTG-3′	53	111
hld-2	5′- TTAGTGAATTTGTTCACTGTGTCGA-3′		
coa-F	5′-AGGTACGTTAATCGGTTTTGG-3′	55	1,586
coa-R	5′-CAGGGTCATCAGGTTGTTCAG-3′		
coa-3	5′-ACCACAAGGTACTGAATCAACG-3′	53	986
coa-4	5′-TGCTTTCGATTGTTCGATGC-3′		
coa-7	5′-GGCTGAGATGGAAGCGATGA-3′	52	608
coa-8	5′-TGACTCGGTTGAGATGGTGC-3′		
clfA-1	5′-AGCGTTAATGCTGCACCTAA-3′	55	1,091
clfA-2	5′-GCGTTACTATCCGTATTTGGTT-3′		

**Table 2:** Biochemical tests.

Test item	result	Test item	result
Glucose	+	Hydrogen sulfide test	−
Maltose	+	Citrate	+
Mannitol	+	MR test	−
Sucrose	−	Indole test	−
Lactose	−	V–P Test	−

(+ indicates positive; - indicates negative).

**Table 3:** Pathogenicity test results of Staphylococcus in BALB/c mice.

Strain	Dose (CFU/g)	Total number	Number of deaths			Total deaths
			0–12 hr	12–24 hr	24–48 hr	
Staphylococcus group	0.6 × 10^8^	10	4	5	1	10
	0.3 × 10^8^	10	2	6	2	10
	1.2 × 10^7^	10	2	5	3	10
	0.6 × 10^7^	10	1	0	0	1
	1.2 × 10^6^	10	0	0	0	0
	1.2 × 10^5^	10	0	0	0	0

Control group	0	10	0	0	0	0

**Table 4:** Detailed results of the antimicrobial susceptibility testing of Staphylococcus.

Abbreviation	Antibiotic	Drug content of paper (*µ*g)	Diameter of the inhibitory zone (mm)	R/I/S
AMP	Ampicillin	10	27	S
CRO	Ceftriaxone	15	0	R
PEN	Penicillin G	10	18	I
CH	Cefradine	30	0	R
CN	Cephalexin	30	23	S
AMC	Amoxicillin	20	29	S
CLR	Clarithromycin	15	0	R
RXM	Roxithromycin	15	0	R
ERY	Erythromycin	15	0	R
CC	Clindamycin	2	0	R
Doxy	Doxycycline	30	16	I
PB	Polymyxin B	300	11	I
RL	Sulfamethoxazole	1.25	0	R
SF	Sulfafurazole	300	0	R
CN	Gentamycin	10	13	I
STR	Streptomycin	10	16	I
ENR	Enrofloxacin	5	16	I
LEV	Levofloxacin	5	17	I
MTZ	Metronidazole	5	0	R
KN	Kanamycin	30	15	I

(S: sensitive; R: resistant; I: moderate sensitivity).

## Data Availability

All data included in this study are available upon request from the corresponding author.
